# DENV-2 Genetic Diversity and Variation in Antibody-Dependent Enhancement in Lao PDR

**DOI:** 10.3390/ijms27198869

**Published:** 2026-10-05

**Authors:** Dalouny Xayavong, Virasack Somoulay, Bouaphanh Khamphaphongphane, Phonepadith Xangxayarath, Hiromu Osako, Kei Yamasato, Motoki Ihara, Jean Claude Balingit, Mya Myat Ngwe Tun, Kouichi Morita, Arata Hidano, Futoshi Hasebe, Yuki Takamatsu

**Affiliations:** 1 Department of Virology, Institute of Tropical Medicine, Nagasaki University, 1-12-4 Sakamoto, Nagasaki 852-8523, Japanjcpbalingit@gmail.com (J.C.B.); moritak@nagasaki-u.ac.jp (K.M.); arata.hidano@nagasaki-u.ac.jp (A.H.); 2 Graduate School of Biomedical Sciences, Nagasaki University, 1-12-4 Sakamoto, Nagasaki 852-8523, Japan; rainbow@nagasaki-u.ac.jp; 3 Program for Nurturing Global Leaders in Tropical and Emerging Communicable Diseases, Nagasaki University, 1-12-4 Sakamoto, Nagasaki 852-8523, Japan; 4 National Center for Laboratory and Epidemiology, Ministry of Health, Vientiane 01002, Laos; 5 Department of Communicable Diseases Control, Ministry of Health, Vientiane 01002, Laos; 6 School of Medicine, Nagasaki University, 1-12-4 Sakamoto, Nagasaki 852-8523, Japan; 7 Department of Tropical Viral Vaccine Development, Institute of Tropical Medicine, Nagasaki University, 1-12-4 Sakamoto, Nagasaki 852-8523, Japan; 8 Dejima Infectious Disease Research Alliance, Nagasaki University, 1-12-4 Sakamoto, Nagasaki 852-8523, Japan; 9 Center for Vaccines Disease and Therapeutic Antibodies for Emerging Infectious Diseases, Shimane University, 1060 Nishikawatsucho, Matsue 690-8504, Japan; 10 Vietnam Research Station, Institute of Tropical Medicine, Nagasaki University, 1-12-4 Sakamoto, Nagasaki 852-8523, Japan

**Keywords:** dengue virus, neutralizing antibody, antibody-dependent enhancement, Lao PDR

## Abstract

All four dengue virus serotypes (DENV-1 to DENV-4) can cause severe and potentially fatal disease. Understanding the interplay between host immune responses and viral genetic diversity is particularly crucial in hyperendemic regions, where serotype shifts frequently occur during outbreaks. In this cross-sectional, surveillance-based study, we analyzed 350 patients with confirmed secondary dengue infection in Lao PDR, with samples collected between 2022 and 2023. Of these, 80 were successfully serotyped by RT-PCR and included in the serotype-specific analyses. Viral genotyping was performed using envelope (E) gene sequencing, and neutralizing antibody (nAb) titers and antibody-dependent enhancement (ADE) activities against all four DENV serotypes were assessed using single-round infectious particles in both FcγRIIA-negative and FcγRIIA-positive BHK-21 cells. The nAb titers were highest against DENV-2, followed by DENV-1, DENV-4, and DENV-3. Nevertheless, DENV-2 exhibited the highest ADE activity, suggesting that high nAb titers may not necessarily prevent ADE. We also documented the co-circulation of DENV-2 Cosmopolitan and Asian I genotypes. Our findings highlight the complex interplay between neutralizing and enhancing antibody responses during secondary infection, particularly in the context of DENV-2 infection. Our results emphasize the need to understand the dynamics of DENV transmission in hyperendemic settings and may inform future vaccine development and evaluation strategies.

## 1. Introduction

Dengue virus (DENV) is a mosquito-borne virus that causes dengue and poses a significant public health concern, particularly in tropical and subtropical regions [1,2]. The dengue virus is a positive-sense, single-stranded RNA virus that encodes three structural proteins: capsid (C), pre-membrane (prM), and envelope (E), and seven non-structural proteins (NS1, NS2A, NS2B, NS3, NS4A, NS4B, and NS5) [3]. Dengue is caused by four distinct DENV serotypes (DENV-1 to DENV-4) that share approximately 65–70% nucleotide sequence identity [4]. Within each serotype, multiple genotypes have been identified, with distributions that vary by region [5].

An estimated 390 million DENV infections occur each year across over 100 countries, of which approximately 500,000 cases require hospitalization [2]. Dengue can range from mild illness without warning signs (dengue without warning signs [DWoWS]) to more severe illness with warning signs (dengue with warning signs [DWWS]), and a small number of patients may develop severe dengue (SD), which involves plasma leakage, bleeding, and organ impairment [6]. It is well documented that SD occurs more frequently in secondary DENV infections involving heterologous serotypes than in primary infections [7,8]. Although infection with one serotype might confer lifelong homologous immunity, it does not necessarily protect against secondary infection with another serotype because pre-existing antibodies may fail to fully neutralize the newly encountered virus. Instead, they can enhance viral infection and contribute to disease severity. This process is primarily explained by antibody-dependent enhancement (ADE), whereby pre-existing subneutralizing or cross-reactive antibodies facilitate enhanced viral entry and replication via Fc receptor-mediated mechanisms [9,10]. Simultaneously assessing neutralizing and enhancing antibody activities may provide a more comprehensive evaluation of the host immune status, as antibodies can both neutralize and enhance DENV infection based on their specificity and concentration. However, other mechanisms also contribute to SD, such as exaggerated cytokine responses and T-cell cross-reactivity, particularly during DENV-2 infection, which is associated with a substantial dengue disease burden and high mortality in tropical regions [11,12,13].

The co-circulation and introduction of these antigenically distinct variants can enhance immune interactions, trigger large outbreaks, and potentially increase disease severity. Studies from Indonesia have indicated that the replacement of genotype IV by genotype I in DENV-1 is associated with an increased risk of dengue hemorrhagic fever (DHF) [14]. Furthermore, evidence from Peru demonstrates that previous infection with DENV-2 may not provide complete protection against homologous reinfections [15]. This complexity poses a major challenge for dengue surveillance and control, and understanding the patterns of serotype co-circulation is crucial in hyperendemic regions.

Dengue remains a major public health concern in Lao PDR, where all four dengue serotypes co-circulate and have caused multiple large epidemics. The first epidemic was initially reported in 1985, followed by major outbreaks of DHF in 1987 in certain provinces [16]. In the 2010 outbreak, the DENV-1 genotype I was the predominant circulating genotype, resulting in 22,890 reported cases and 46 deaths [17,18]. This was followed by the predominance of DENV-3 genotype II during the 2013 outbreak, which caused 48,772 reported cases and 95 deaths. In 2017, DENV-4 genotype I predominated, with 11,067 cases and 14 deaths. Subsequently, DENV-2, including the Cosmopolitan and Asian I genotypes, became the dominant circulating genotype in 2019, accounting for 39,901 cases and 76 deaths [19,20,21]. These successive shifts in serotype and genotype dominance, against a background of sustained co-circulation, increase the risk of secondary infections, which are strongly associated with severe dengue and raise significant concerns about ADE. Recent national surveillance data further indicate that dengue incidence in 2022 and 2023 exceeded the five-year median [22]. These outbreaks were accompanied by continued shifts in circulating serotypes and genotypes, highlighting the dynamic evolution of DENV transmission in Lao PDR. The introduction or shift in a DENV serotype or genotype may contribute to large outbreaks by altering population immunity and viral transmission dynamics, potentially influencing disease severity. However, the virological and immunological factors underlying these outbreaks remain poorly understood. This study therefore aimed to investigate the molecular epidemiology of DENV in Lao PDR through phylogenetic analysis and to evaluate immune correlates, including neutralizing antibody responses and ADE activity, to better understand DENV genetic diversity and functional antibody responses in Lao PDR. These findings may help strengthen dengue surveillance, outbreak preparedness, and future vaccine strategies.

## 2. Results

### 2.1. Patient Characteristics

This study evaluated serum samples from 350 laboratory-confirmed DENV infection cases, identified by NS1 antigen detection, IgM testing, and/or RT-PCR, which were classified as secondary infections and collected through the national surveillance system in Lao PDR during 2022–2023, as previously described [23]. The median age of the patients was 22 years old (interquartile range [IQR], 15–34 years), indicating that secondary infections occurred primarily among young adults during this period. The median time between symptom onset and sample collection was 4 days (IQR, 3–5 days). Females accounted for a higher proportion of cases than males, with a female-to-male ratio of 1.41:1. Cases were categorized as DWoWS (*n* = 130), DWWS (*n* = 140), or SD (*n* = 25), whereas 55 patients were not categorized. Serotyping by RT-PCR was successful for 80 cases, including 28 DENV-1 infections and 52 DENV-2 infections. No DENV-3 or DENV-4 infections were detected during the study period (Table 1).

### 2.2. Neutralizing Antibody Titers and Infection-Enhancing Antibodies in Secondary DENV Infections

**Figure 1:**
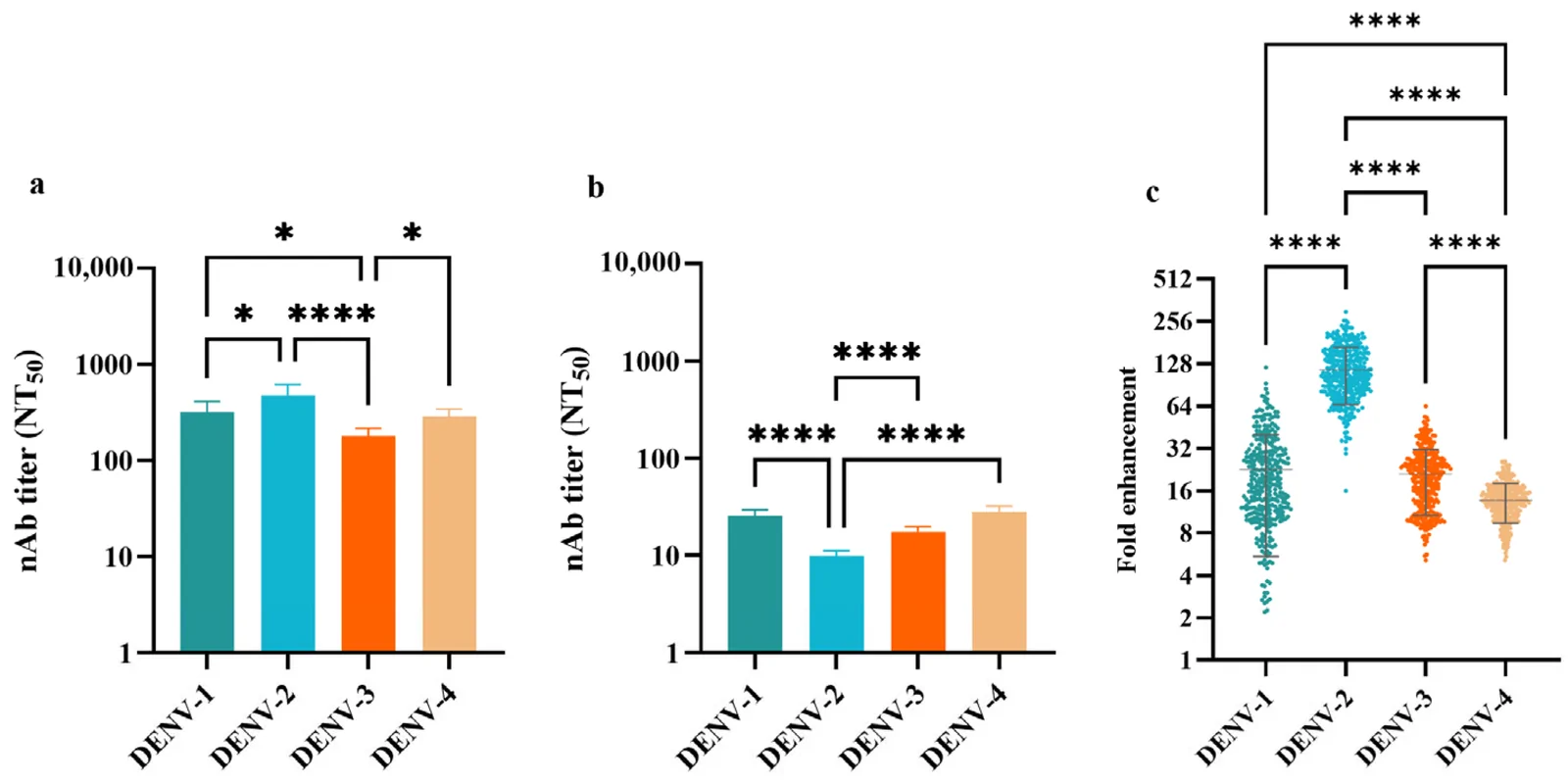
Neutralizing and antibody-dependent enhancement profiles in secondary dengue infection. (**a**,**b**) Serotype-specific neutralizing antibody (nAb) titers measured in BHK-21 cells with and without human FcγRIIA, presented as geometric means with 95% confidence intervals (CIs). (**c**) Antibody-dependent enhancement (ADE) activity by serotype, presented as the median with interquartile range (IQR). Statistical comparisons were performed using the Friedman test. *p* > 0.05 (not shown); * *p* < 0.05 and **** *p* < 0.0001.

To elucidate pre-existing immunity, we evaluated neutralizing antibody titers and infection-enhancing activity against DENV-1–4 in sera from 350 individuals using FcγRIIA-negative and FcγRIIA-positive BHK-21 cells. For SRIP production, we employed DENV genotypes currently circulating in the Lao PDR: DENV-1 genotype I, DENV-2 Cosmopolitan genotype, DENV-3 genotype II, and DENV-4 genotype I. In FcγRIIA-negative BHK-21 cells, neutralizing antibody titers differed significantly among the four DENV serotypes (Friedman statistic = 38.9, *p* < 0.0001). Neutralizing activity was highest against DENV-2 (GMNT50 = 476.5, 95% CI: 369.8–613.9), followed by DENV-1 (GMNT50 = 317.5, 95% CI: 244.5–412.3), DENV-4 (GMNT50 = 284.1, 95% CI: 235.4–343.0), and DENV-3 (GMNT50 = 179.5, 95% CI: 148.5–216.9) (Figure 1a). In FcγRIIA-positive BHK-21 cells, neutralizing antibody titers also differed significantly among the four serotypes (Friedman statistic = 175.2, *p* < 0.0001). Neutralizing titers were lower across all serotypes: DENV-4 (GMNT50 = 27.84, 95% CI: 23.7–32.6), DENV-1 (GMNT50 = 25.37, 95% CI: 21.7–29.6), DENV-3 (GMNT50 = 17.6, 95% CI: 15.6–19.9), and DENV-2 (GMNT50 = 9.9, 95% CI: 8.9–11.2) (Figure 1b). The overall serotype comparison was significant (Friedman statistic = 679.6, *p* < 0.0001), with DENV-2 showing significantly higher infection-enhancing activity (median = 105.1, IQR: 78.8–149.7) than the other serotypes (*p* < 0.0001) (Figure 1c).

**Figure 2:**
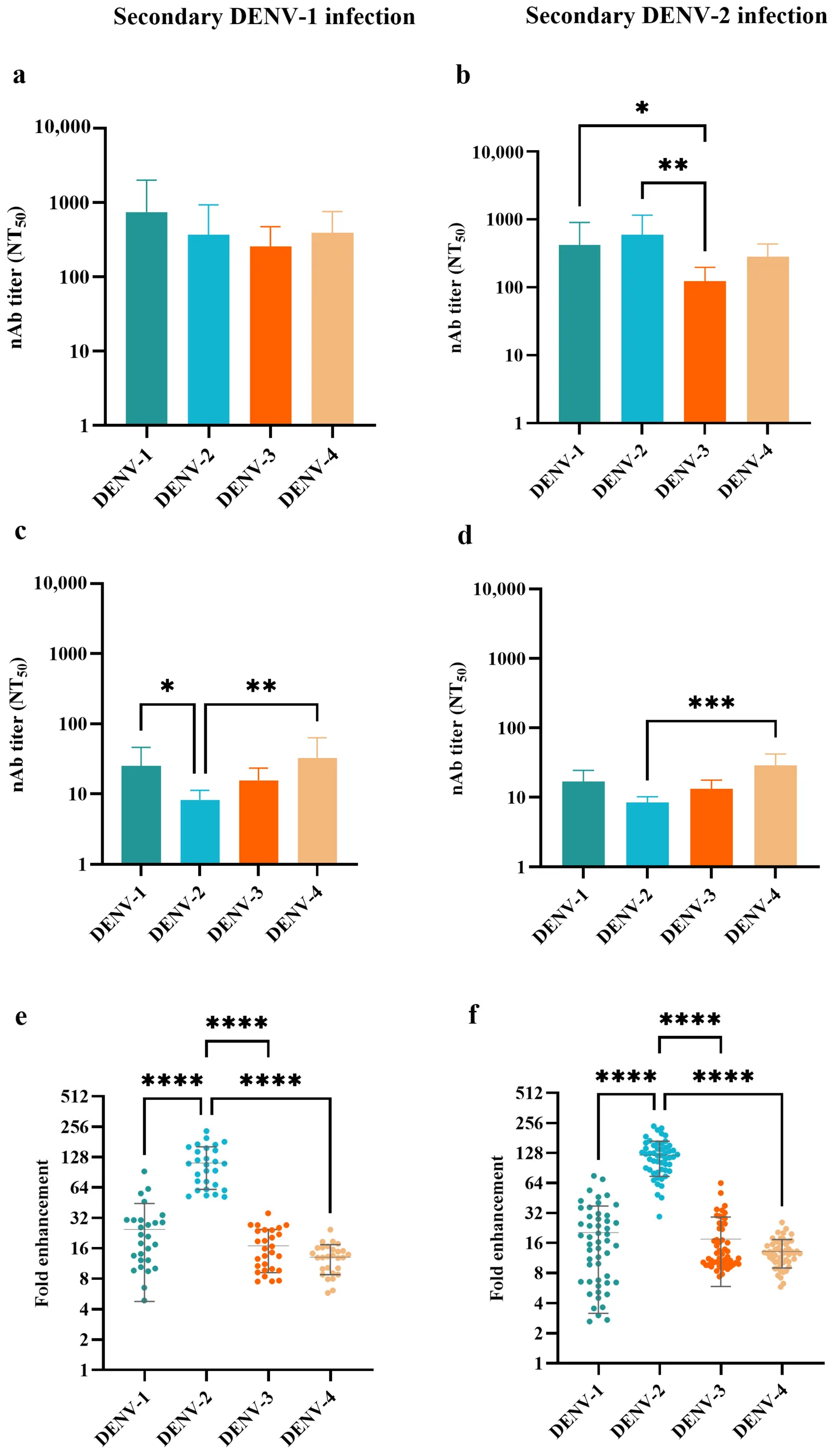
Comparison of nAb and fold-enhancement levels in secondary DENV-1 and DENV-2 infections. Serotype-specific neutralizing antibody (nAb) titers in BHK-21 cells with and without human FcγRIIA and corresponding fold-enhancement values for serum samples from individuals with secondary DENV-1 (**a**,**c**,**e**) and DENV-2 (**b**,**d**,**f**) infections. Neutralizing antibody titers are presented as geometric means with 95% CIs. Antibody-dependent enhancement (ADE) activity by serotype is presented as the median with interquartile range (IQR). Statistical significance was assessed using the Friedman test. *p* > 0.05 (not shown); * *p* < 0.05; ** *p* < 0.01; *** *p* < 0.001; and **** *p* < 0.0001.

Among the samples analyzed in Figure 1, we further analyzed a subset of individuals with secondary DENV-1 (*n* = 28) and DENV-2 (*n* = 52) infection for serotype-specific nAb titers and ADE activity against all four DENV serotypes. In FcγRIIA-negative BHK-21 cells, among individuals with secondary DENV-1 infection, nAb titers did not differ significantly across the four DENV serotypes (Friedman statistic = 4.6, *p* = 0.20). The highest nAb GMNT50 was observed against DENV-1 (GMNT50 = 742.5, 95% CI: 274.3–2010), followed by DENV-2, DENV-4, and DENV-3. Among individuals with secondary DENV-2 infection, nAb titers differed significantly across the four DENV serotypes (Friedman statistic = 94.6, *p* < 0.0001). The nAb titers were significantly higher against DENV-2 than against DENV-3 (*p* < 0.01), and titers against DENV-1 and DENV-4 were lower than those against DENV-2 but higher than those against DENV-3. These patterns were consistent with the epidemiological data showing the predominance of DENV-1 and DENV-2 (Figure 2a,b). In FcγRIIA-positive BHK-21 cells, nAb titers differed significantly across the four DENV serotypes among individuals with secondary DENV-1 infection (Friedman statistic = 17.3, *p* = 0.0006) and among individuals with secondary DENV-2 infection (Friedman statistic = 23.3, *p* < 0.0001). The nAb titers against DENV-2 were substantially reduced in both the secondary DENV-1 infection group (GMNT50 = 8.20, 95% CI: 5.9–11.2) and the secondary DENV-2 infection group (GMNT50 = 8.40, 95% CI: 6.9–10.2) compared with those in the FcγRIIA-negative cells (Figure 2c,d). Fold enhancement differed significantly across the four DENV serotypes in both the secondary DENV-1 infection group (Friedman statistic = 56.1, *p* < 0.0001) and the secondary DENV-2 infection group (Friedman statistic = 94.6, *p* < 0.0001). The highest mean fold enhancement was against DENV-2 (median = 108.6, IQR: 62.9–154.7 for the DENV-1 group; median = 120.5, IQR: 86.0–150.5 for the DENV-2 group), followed by DENV-1 (median = 19.4, IQR: 10.8–30.3; median = 15.9, IQR: 6.5–28.9), DENV-3 (median = 15.2, IQR: 9.8–23.9; median = 12.0, IQR: 9.9–24.3), and DENV-4 (median = 13.0, IQR: 9.3–15.9; median = 12.7, IQR: 10.3–15.7) (Figure 2e,f).

### 2.3. Phylogenetic Analysis Based on the DENV-2 Envelope Gene

**Figure 3:**
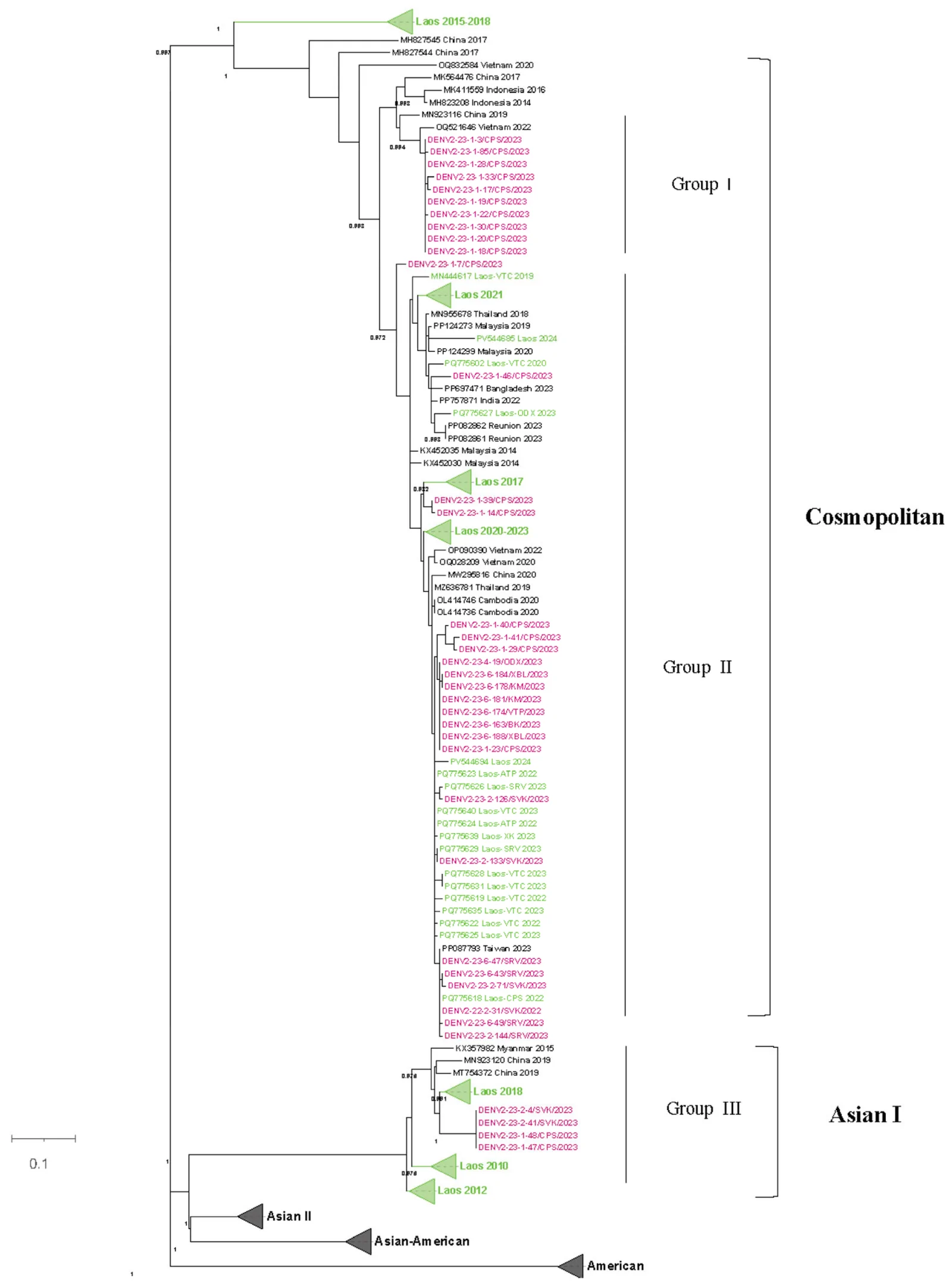
DENV-2 phylogenetic tree and genotype classification. A maximum-likelihood phylogeny was inferred using the general time-reversible model with invariant sites and gamma distribution (GTR + I + G) with 1000 bootstrap replicates. Sequences generated in this study are highlighted in pink, and previously published Lao sequences are shown in green.

Phylogenetic analysis was performed using the 1485 bp envelope (E) gene. Reference sequences representing DENV-2 genotypes I–V were retrieved from the NCBI database, together with previously reported sequences from the Lao PDR. We successfully obtained full-length E gene sequences from 37 DENV-2 strains collected between 2022 and 2023. These sequences clustered into two genotypes, Cosmopolitan and Asian I (Figure 3 and Figure S1), and were further grouped into three phylogenetic clusters.

Group 1 comprised 10 sequences, including those from DWoWS, DWWS, and SD cases, collected in Champasack Province. These sequences formed a single clade with a Vietnamese strain collected in 2022. Group 2 included 23 sequences that were closely related to Lao strains collected between 2017 and 2023, as well as strains from neighboring countries, including Vietnam, Thailand, China, and Cambodia. Group 3 consisted of four sequences from Savannakhet and Champasack provinces that belonged to the Asian I genotype and were closely related to Lao strains collected in 2018. These findings indicate the concurrent circulation of two DENV-2 genotypes (Cosmopolitan and Asian I) in Lao PDR.

### 2.4. Genotype-Specific Differences in ADE Activity

**Figure 4:**
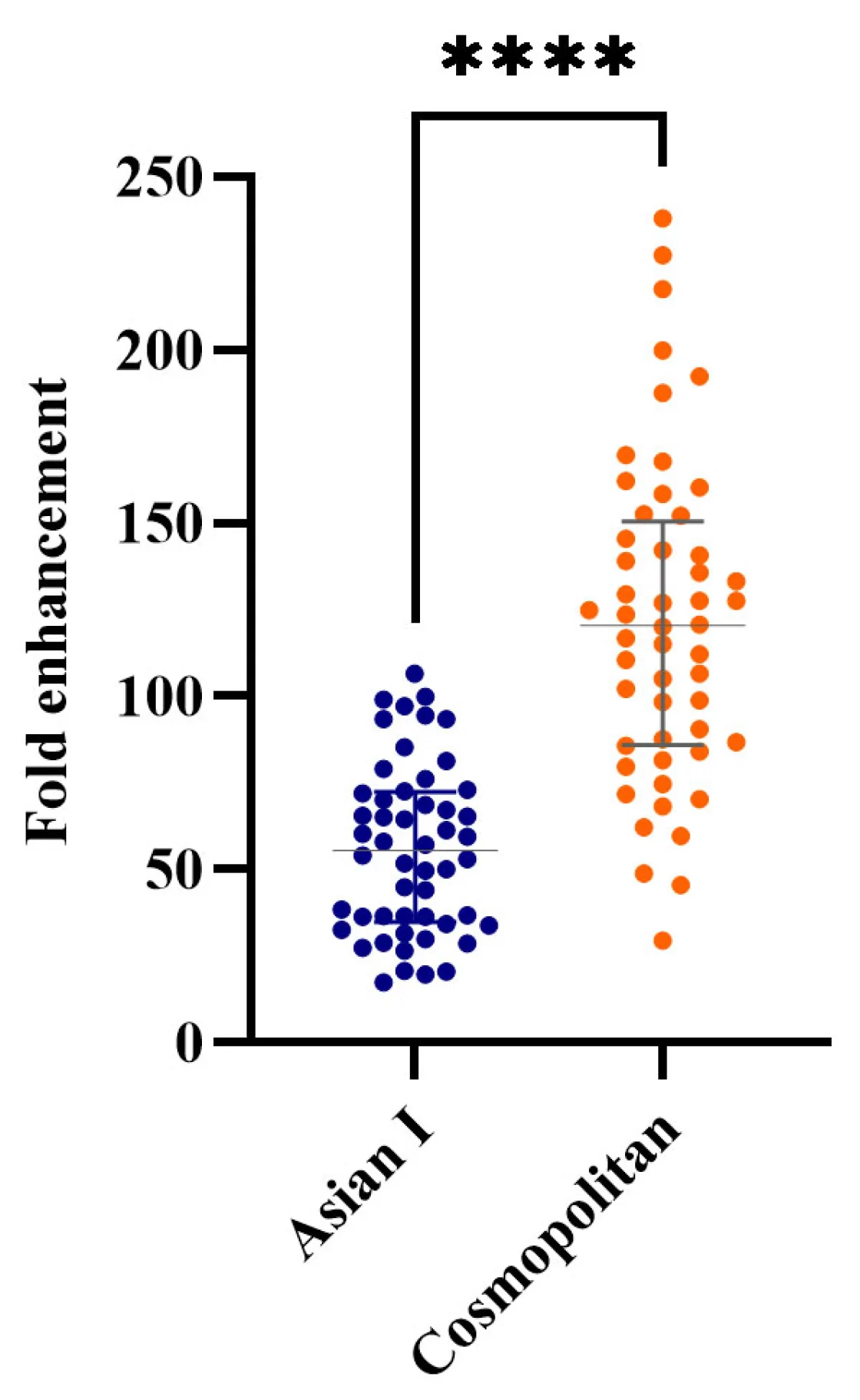
ADE activity against Asian I and Cosmopolitan DENV-2 genotypes in secondary infections. Fold enhancement (ADE titers) against Asian I and Cosmopolitan SRIPs induced by serum samples from secondary DENV-2 infections (*n* = 52). Data are presented as the median with interquartile range (IQR). Paired comparisons were performed using the Wilcoxon matched-pairs signed-rank test. The geometric mean ratio (Cosmopolitan/Asian I) and its 95% confidence interval were calculated from paired fold enhancement ratios on the logarithmic scale and back-transformed to the original scale. **** *p* < 0.0001.

To examine genotype-specific differences in ADE activity, we compared serum samples from DENV-2-infected patients using SRIPs derived from the historically circulating DENV-2 Asian I genotype and the currently circulating DENV-2 Cosmopolitan genotype. Serum samples from DENV-2-infected patients demonstrated significantly greater ADE activity against the Cosmopolitan genotype than against the Asian I genotype (Wilcoxon matched-pairs signed-rank test, *p* < 0.0001), with a geometric mean ratio (Cosmopolitan/Asian I) of 1.42 (95% CI: 1.32–1.51) (Figure 4).

## 3. Discussion

These findings highlight the complex interplay between neutralizing and enhancing antibodies, particularly in the context of DENV-2 infection in the Lao population during 2022 and 2023. We found that DENV-2 exhibited both high neutralizing activity and potent ADE activity. In addition, phylogenetic analysis revealed genetic diversity within DENV-2, with co-circulation of the Cosmopolitan and Asian I genotypes. These results underscore the importance of both host immune responses and viral genetic diversity in shaping dengue transmission dynamics and disease outcomes.

Neutralizing antibodies (nAbs) are central to protective immunity against DENV [24]. In FcγRIIA-negative BHK-21 cells, sera from individuals with secondary infections showed the highest neutralizing activity against DENV-2, followed by DENV-1, suggesting prior exposure to DENV-1 and/or DENV-2. Historically, outbreaks in Lao PDR have been driven mainly by DENV-1 and DENV-2, with temporal shifts in their relative dominance [21,25]. In contrast, in FcγRIIA-positive BHK-21 cells, nAb titers against DENV-2 were the lowest among the four serotypes. Because FcγRIIA-positive BHK-21 cells capture the net balance between neutralization and ADE, our findings indicate that sera with high neutralizing activity may also contain strongly enhancing antibodies. The detection of neutralization in the absence of FcγR engagement may not reflect true protective neutralization, as these assays do not account for FcγR-mediated viral entry pathways and may therefore overestimate functional neutralizing capacity [26]. This observation is particularly noteworthy in dengue-endemic countries. Serotype-specific nAb profiles provide insights into natural immunity, help identify recent shifts in circulating serotypes, and may guide the development of effective vaccines and therapeutics against dengue.

ADE can be mediated by serotype-cross-reactive antibodies, low-affinity antibodies, or antibodies targeting specific epitopes, including those in the prM or E proteins [24,27,28]. In this study, we observed a significant enhancement of DENV-2 infection despite the presence of high nAb titers against the same serotype (Figure 1c). This finding suggests that a subset of antibodies induced by prior infections was cross-reactive and exhibited incomplete neutralization against the currently infecting serotype, thereby facilitating viral entry. Human anti-prM antibodies are highly cross-reactive among dengue serotypes and, as previously reported, do not neutralize infection even at high concentrations but instead potently promote ADE [27]. Many patients with DENV-2 infection had both high neutralizing titers and substantial enhancement activity, as shown in Figure 2. One possible explanation for the high DENV-2 nAb titers is prior exposure to DENV-2 strains, including the Asian I genotype, which predominated in Lao PDR until approximately a decade ago. However, individual histories of previous DENV infections and information on previously infecting genotypes were unavailable; therefore, this hypothesis could not be directly evaluated. The Cosmopolitan genotype has been detected in Lao PDR since at least 2015, and the Cosmopolitan and Asian I genotypes subsequently co-circulated. However, regional surveillance conducted from 2020 to 2023 did not detect the Asian I genotype [29], suggesting a shift toward the predominance of the Cosmopolitan genotype. In our study, ADE activity was significantly greater against the Cosmopolitan genotype than against the Asian I genotype (Figure 4). However, the clinical significance of this genotype-specific difference in ADE activity remains unclear. Previous data from the Philippines indicate that prior infection with DENV-2 Asian II can elicit antibodies that enhance infection with the Cosmopolitan genotype [28]; whether such genotype-related differences in ADE are specific to DENV-2 or represent a broader cross-serotype phenomenon requires further investigation.

Our previous studies by Xayavong D et al. (2026) indicated that DENV-2 was associated with SD during this period [23]. The present findings further support this observation by showing that DENV-2 exhibited higher ADE activity than the other serotypes. In the exploratory multivariable logistic regression analysis, DENV-2 infection had an adjusted odds ratio (aOR) of 6.18 for DWWS/SD compared with DWoWS (95% CI: 1.75–26.0, *p* = 0.007) (Figure S3). However, disease severity is likely influenced by multiple factors, including host and viral factors, such as viral fitness, host genetic susceptibility, T-cell responses, cytokine dysregulation and viral load [11,12,30]. Due to the aforementioned limitations of the logistic regression analysis, careful interpretation is required. One possible explanation for the greater clinical severity observed among DENV-2 infections is viral genetic variation, including variation within the Cosmopolitan genotype, which may influence viral replication and pathogenicity [31,32]. Supporting this, Samune et al. demonstrated that structural and nonstructural proteins from the DENV-2 Cosmopolitan genotype increased viral replication and disease severity in vitro and in vivo [33]. Thus, the higher intrinsic virulence of the DENV-2 Cosmopolitan genotype cannot be excluded.

All DENV-1 E-gene sequences obtained in this study clustered within genotype I, consistent with previous reports of long-term DENV-1 circulation in Lao PDR (Figure S4). In contrast, DENV-2 E gene sequences segregated into three phylogenetic groups belonging to the Cosmopolitan and Asian I genotypes. This finding is consistent with reports from Vietnam describing a shift in circulating DENV-2 strains from the Asian I genotype to the Cosmopolitan genotype during the same period [34]. Notably, the Asian I genotype identified in this study was detected in Savannakhet and Champasack provinces, both of which are high-burden dengue areas that share borders with Thailand, where the Asian I genotype continues to circulate [35,36]. This observation may be associated with cross-border population movement and suggests the possible co-circulation of multiple genotypes in these regions. These findings highlight the importance of continuous molecular surveillance for monitoring genotype shifts and DENV transmission dynamics.

Notably, multiple amino acid substitutions were identified, including the V347I substitution (valine-to-isoleucine substitution) (Figure S5), which was distinct from historical DENV-2 sequences from Lao PDR and was observed in association with one phylogenetic group. The V347I substitution was also reported during the 2022–2023 outbreak in Pakistan [37]. Although mutations in domain III (EDIII) have been associated with changes in viral infectivity and immune evasion, the biological significance of V347I remains unclear [38,39]. Further experimental studies are needed to determine whether V347I or other substitutions identified in this study affect viral replication, infectivity, immune evasion, or pathogenicity. Although our analysis focused on the E gene, comprehensive whole-genome sequencing will be needed to fully resolve the recent evolutionary trajectory of DENV in Lao PDR.

To our knowledge, this is the first study to characterize serotype- and genotype-specific ADE activity in the Lao population. However, confirming and refinement of our conclusions will require larger sample sizes and more detailed infection histories. This study had several limitations. First, only DENV-1 and DENV-2 were detected; therefore, comparisons with DENV-3 and DENV-4 remain unresolved. In addition, some samples could not be serotyped, potentially because of their low viral loads. Because DENV viral loads can be influenced by the infecting DENV genotype and pre-existing immune status, certain DENV genotypes may have been underdetected in this study. The availability of samples suitable for RT-PCR serotyping and sequencing may also have introduced selection bias, as included and non-included samples may have differed systematically. Second, serum samples were collected at a single time point, preventing longitudinal assessment of antibody dynamics throughout infection. Detailed individual histories of previous DENV infections and information on previously infecting genotypes were unavailable. Third, only the envelope gene was sequenced rather than the whole genome. Consequently, genetic variations in other genomic regions that may contribute to viral fitness, pathogenicity, or viral transmission could not be assessed. In addition, viral-load measurements were not available, limiting our ability to evaluate the relationship among viral load, immune response, and disease severity. The limited number of severe dengue cases, particularly among serotyped samples, may have reduced the precision of the severity analysis. Residual confounding in the observed relationships among DENV serotype, ADE, and disease severity cannot be excluded. Furthermore, because samples were obtained through the national dengue surveillance system, the study population may not fully represent all dengue infections in the community, particularly asymptomatic infections and cases that did not present to healthcare facilities. Therefore, healthcare-seeking and sample-availability biases cannot be excluded. Although the Cosmopolitan genotype was associated with higher ADE activity than the Asian I genotype in secondary DENV-2 infections, this observational study cannot determine whether the genotype shift directly contributed to enhanced ADE or increased disease severity. Larger prospective longitudinal studies are needed to clarify these findings. In addition, experimental characterization of the distinctive DENV-2 genetic variants identified in this study remains necessary.

## 4. Materials and Methods

### 4.1. Ethical Considerations

The study was approved by the National Ethics Committee for Health Research, Lao PDR (No. 48/NECHR) and the Institutional Review Board of the Institute of Tropical Medicine, Nagasaki University (No. 240215305). All sample information was anonymized, and personal identifiers were removed to protect patient confidentiality; only coded data were used for analysis. Samples were collected as part of a national surveillance program for public health purposes; therefore, the requirement for individual written informed consent was waived for this study. All experiments were conducted according to the relevant guidelines and regulations.

### 4.2. Study Population and Sample Collection

A cross-sectional study was conducted as part of the National Surveillance System in Lao PDR during 2022 and 2023. Samples were obtained from 13 of the 18 provinces across the country, as described in a previous study [23]. Briefly, 1098 serum samples from patients suspected of having dengue were retrieved. Among them, 615 samples were laboratory-confirmed as DENV-positive by at least one of the following assays: NS1, IgM, and/or RT-PCR. Samples that tested negative in all three assays were classified as DENV-negative. Primary and secondary DENV infections were classified using in-house DENV IgM/IgG ELISAs [40,41,42].

Two independent analyses were performed in this study (Figure S2). For the immunological analyses, 350 samples from patients with secondary infection (IgG titer ≥ 29,000) were selected. Of these, 80 RT-PCR-serotyped samples (28 DENV-1 and 52 DENV-2) were included in the serotype-specific analyses. Separately, for the molecular epidemiological analyses, complete envelope (E) gene sequences were obtained from 32 DENV-1 and 37 DENV-2 RT-PCR-serotyped samples selected from the laboratory-confirmed DENV-positive cases.

Clinical manifestations of dengue were classified according to the 2009 World Health Organization (WHO) criteria, which include dengue without warning signs (DWoWS), dengue with warning signs (DWWS), and severe dengue (SD).

### 4.3. Classification of Secondary DENV Infection

An in-house DENV IgG indirect ELISA was performed to determine IgG titers, and optical density (OD) values were measured at 495 nm. An IgG titer ≥ 29,000 was considered indicative of a secondary infection, whereas a titer < 29,000 was classified as a primary infection [41,43].

### 4.4. Envelope Gene Sequencing

PCR amplification of the DENV envelope (E) gene was performed using KOD One™ PCR Master Mix (2×) (Toyobo, Osaka, Japan), as previously described. Sequencing of the E gene region was performed using the BigDye™ Terminator v3.1 Cycle Sequencing Kit (Thermo Fisher Scientific, Austin, TX, USA), following the manufacturer’s instructions, on a 3500 Genetic Analyzer (Applied Biosystems, Tokyo, Japan). The complete E gene was amplified with two flanking primers, and four additional primers were used to generate overlapping fragments for sequencing, as previously described [44].

### 4.5. Phylogenetic Analysis

The complete E gene sequences (1485 nucleotides) generated in this study were assembled and analyzed using CodonCode Aligner (CCA) v12.0.1 (CodonCode Corporation, Dedham, MA, USA). Newly generated sequences were aligned with previously reported sequences from Lao PDR and additional sequences retrieved from NCBI. Representative sequences were selected using CD-HIT-EST v4.8.1 with a nucleotide sequence identity threshold of 90% [45]. Multiple-sequence alignments of DENV-1 and DENV-2 were performed using MAFFT v7.515 [46]. The best-fitting nucleotide substitution model was determined using the model-selection function in MEGA v12.0 based on the Bayesian Information Criterion (BIC). Maximum-likelihood phylogenetic trees were constructed using the general time-reversible model with a proportion of invariant sites and gamma-distributed rate variation (GTR + I + G), with 1000 bootstrap replicates [47]. All sequences were submitted to GenBank under accession numbers PZ177878-PZ177909 for DENV-1 and PZ178842-PZ178878 for DENV-2 (Table S1).

### 4.6. Cells

Human embryonic kidney 293T (HEK-293T) cells obtained from the American Type Culture Collection (ATCC; CRL-3216) were maintained in high-glucose Dulbecco’s modified Eagle’s medium with L-glutamine (DMEM; Fujifilm Wako, Osaka, Japan) supplemented with 10% fetal bovine serum (FBS; Biowest, Bradenton, FL, USA), 1× MEM non-essential amino acids (Gibco, Grand Island, NY, USA), and 100 U/mL penicillin–streptomycin (Sigma-Aldrich, Tokyo, Japan). Baby hamster kidney (BHK21) cells were obtained from the European Collection of Authenticated Cell Cultures (ECACC; 85011433) and maintained in Eagle’s minimum essential medium (EMEM; Fujifilm Wako, Osaka, Japan) supplemented with 10% fetal bovine serum (FBS) and 100 U/mL penicillin–streptomycin. Human FcγRIIA-expressing BHK21 cells, generated using a lentiviral vector (Osako et al., manuscript in preparation), were maintained in EMEM supplemented with 10% FBS, 100 U/mL penicillin–streptomycin, and 100 µg/mL G418 sulfate (Fujifilm Wako, Osaka, Japan). All cell lines were cultured at 37 °C in a humidified incubator with 5% CO_2_. 

### 4.7. Production and Titration of Single-Round Infectious Particles (SRIPs)

A yellow fever virus (YFV) subgenomic replicon plasmid derived from the YFV 17D vaccine strain (X03700), containing the NanoLuc luciferase reporter gene (pCMV-YFV-nluc-rep), and an expression plasmid encoding the mature YFV 17D capsid protein consisting of 100 amino acids (pCAG-YF-C) were constructed as described previously [48]. To generate DENV prM/E expression plasmids, we used the prM and E regions of the following strains: DENV-1 D1/Hu/Saitama/NIID100/2014 strain, genotype I (LC011945; Japan); DENV-2 36_DENV-2 Cosmopolitan genotype (MN083232; Sri Lanka); DENV-2 ThNH-52/93 Asian I genotype (AF022436; Thailand); and DENV-3 CNR_15418 genotype II (MH888332; Thailand). The DENV-4 prM/E expression plasmid was synthesized from synthetic DNA (GENEWIZ, South Plainfield, NJ, USA) based on the nucleotide sequence of MH888334 (GenBank, Bethesda, MD, USA). HEK293T cells in 10 cm dishes were co-transfected with 2.5 µg of replicon plasmid, 1.25 µg of capsid expression plasmid, and 1.25 µg of prM/E expression plasmid in Opti-MEM (Gibco, Grand Island, NY, USA) using polyethylenimine HCl Max, MW 40,000 (Polysciences, Warrington, PA, USA), as described previously. After 5 h, the culture medium was replaced with fresh medium. At 2 days post-transfection, the culture supernatants were harvested and stored at −80 °C as SRIPs for subsequent experiments [49]. For SRIP titration, BHK21 cells were seeded in 96-well plates and incubated overnight. Serial dilutions of SRIPs were prepared in EMEM supplemented with 2% FBS, and each dilution was tested in triplicate. Diluted SRIPs were added to the cell monolayers and incubated at 37 °C in 5% CO_2_ for 5 h. The inoculum was then replaced with fresh medium, and the plates were incubated at 37 °C in 5% CO_2_ for 2 days. Luciferase activity was measured using the NanoGlo^®^ Luciferase Assay System (Promega, Madison, WI, USA) and quantified with a GloMax Explorer plate reader (Promega, Madison, WI, USA). The working dilution for neutralization and ADE assays was defined as the highest SRIP dilution that produced at least a 100-fold higher luciferase signal than the cell-only control in all four replicate wells [50]. 

### 4.8. Neutralization and ADE Assays Using SRIPs

Neutralization assays were performed using FcγRIIA-negative and FcγRIIA-positive BHK21 cells, whereas ADE assays were conducted exclusively using FcγRIIA-positive BHK21 cells. Cells were seeded in 96-well plates at a density of 6.0–8.0 × 10^4^ cells/well and incubated overnight. Serum samples were heat-inactivated at 56 °C for 30 min before testing. For neutralization and ADE assays, the sera were serially diluted fourfold in EMEM supplemented with 2% FBS (from 1:10 to 1:163,840) and mixed with SRIPs at a 1:1 (*v*/*v*) ratio. The serum–SRIP mixtures were incubated at 37 °C for 1 h and then added to the cell monolayers. The plates were incubated at 37 °C in 5% CO_2_ for 5 h, after which the inoculum was replaced with fresh medium. The cells were then incubated at 37 °C in 5% CO_2_ for 2 days. Each plate contained SRIP-only and cell-only controls, and all serum samples were tested in duplicate. Luciferase activity was measured 2 days post-infection using the NanoGlo Luciferase Assay System and read on a GloMax Explorer plate reader (Promega, Madison, WI, USA). Neutralizing antibody (nAb) titers were expressed as the serum dilution that reduced the luciferase signal by ≥50% (NT50). Fold enhancement values were plotted against the logarithm of the reciprocal serum dilution. The reduction in luciferase signal and fold-enhancement values were calculated as described previously [28,50].

### 4.9. Statistical Analysis

Serotype-specific neutralizing antibody (nAb) titers in BHK-21 cells with and without human FcγRIIA are presented as geometric means with 95% CIs, whereas fold-enhancement values are presented as the median with interquartile range (IQR). Comparisons of nAb titers and fold-enhancement values against the four DENV serotypes were performed using the Friedman test, followed by Dunn’s multiple-comparisons test for pairwise comparisons between serotypes. Fold enhancement values between the DENV-2 Asian I and Cosmopolitan genotypes were compared using the Wilcoxon matched-pairs signed-rank test. The geometric mean ratio (Cosmopolitan/Asian I) and its 95% confidence interval were calculated from paired fold enhancement ratios on the logarithmic scale and back-transformed to the original scale. All statistical analyses and figure generation were performed using GraphPad Prism 10 software (GraphPad Software Inc., San Diego, CA, USA). A two-sided *p*-value of < 0.05 was considered statistically significant. Detailed explanations are provided in the Supplementary Methods.

## 5. Conclusions

Our study demonstrates the concurrent circulation of multiple DENV serotypes and genotypes and identifies virological and immunological characteristics that may contribute to dengue pathogenesis. We characterized serotype- and genotype-specific ADE activity in Lao PDR and observed higher ADE activity against DENV-2 under the experimental conditions evaluated, while exploratory analyses suggested an association between DENV-2 infection and greater clinical severity. These findings should be interpreted cautiously because of the cross-sectional design, limited number of severe cases, and potential residual confounding. However, the relationship between ADE activity and clinical severity remains unclear. Therefore, longitudinal studies are needed to validate these findings and clarify the underlying biological mechanisms. We also documented the co-circulation of DENV-2 Cosmopolitan and Asian I genotypes. Taken together, these findings highlight the importance of sustained genomic and immunological surveillance to inform vaccine introduction, optimize vector control, and strengthen preparedness for future dengue epidemics in Lao PDR.

## Figures and Tables

**Table 1:** Descriptive characteristics and DENV classification of 350 patients with confirmed secondary dengue infection.

Characteristic	DWoWS (*n* = 130)	DWWS (*n* = 140)	SD (*n* = 25)	Not Defined (*n* = 55)	Total (*n* = 350)
**Age (Median IQR)**	22 (15–33)	22 (15–32.5)	22 (15–35)	25 (16.5–36.5)	22 (15–34)
**Onset day (Median IQR)**	3 (2–4)	4 (3–5)	4.5 (3.75–5)	4 (3–5.5)	4 (3–5)
**Gender**						
**Female**	75	85	16	29	205
**Male**	55	55	9	26	145
Serotype (n = 80) *						
**DENV-1**	18	7	1	2	28
**DENV-2**	12	18	6	16	52

* Serotype classification was based on successfully serotyped samples (n = 80).

## Data Availability

The original contributions presented in this study are included in the article and Supplementary Materials. Further inquiries can be directed to the corresponding author.

## Supplementary Materials

The following supporting information can be downloaded at: https://www.mdpi.com/article/10.3390/ijms27198869/s1.

## Abbreviations

The following abbreviations are used in this manuscript:

Lao PDR	Lao People’s Democratic Republic
WHO	World Health Organization
DWoWS	Dengue without warning signs
DWWS	Dengue with warning signs
SD	Severe dengue
DHF	Dengue hemorrhagic fever
ELISA	Enzyme-linked immunosorbent assay
NS1	Nonstructural protein 1
IgM	Immunoglobulin M
IgG	Immunoglobulin G
ADE	Antibody-dependent enhancement
nAb	Neutralizing antibody
YFV	Yellow fever virus
