# Supplementary material for: DENV-2 Genetic Diversity and Variation in Antibody-Dependent Enhancement in Lao PDR
Source: Int J Mol Sci. 2026 Oct 5;27(19):8869. doi: 10.3390/ijms27198869 (PMC13664945; doi:10.3390/ijms27198869)
Supplement: Supplementary file 1 [file ijms-27-08869-s001.zip › ijms-4445965-supplementary.pdf]

S1. Supplementary Materials

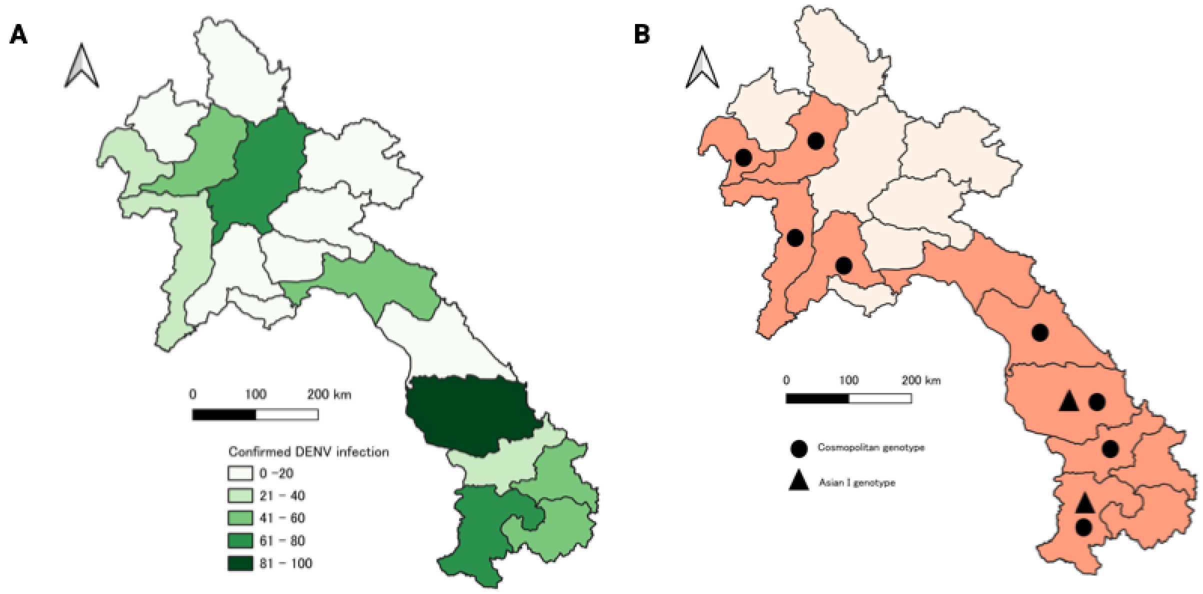

Figure S1. Geographic distribution of DENV and DENV-2 confirmed serum samples. (A) Distribution of the 350 serum samples across 13 provinces; (color intensity proportional to the number of samples. (B) Distribution of DENV-2-positive samples across 11 provinces, highlighted in orange. Black circles indicate the detection of the Cosmopolitan genotype, whereas black triangles indicate the detection of the Asian I genotype.

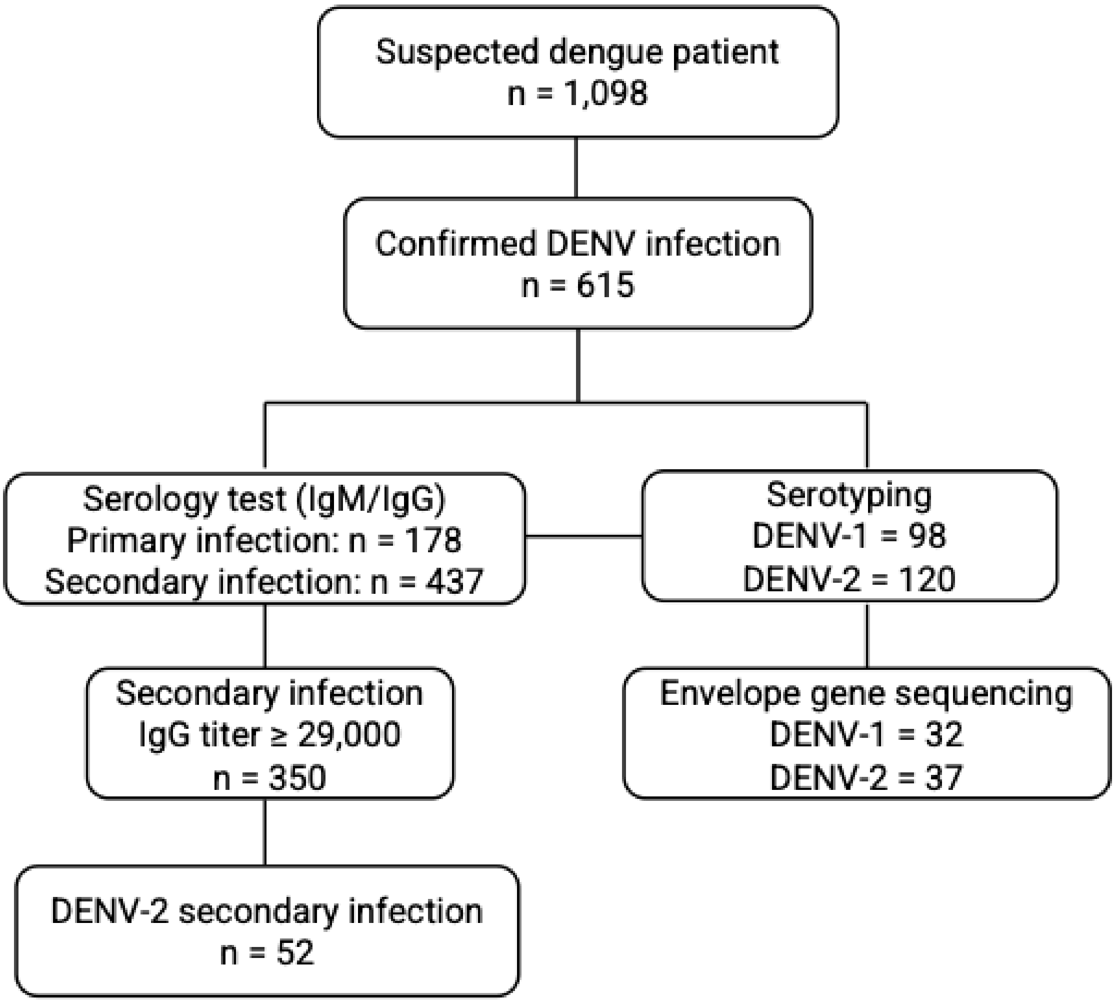

Figure S2. Enrollment and confirmed of DENV infection. A total of 1,098 serum samples were collected from patients with suspected dengue. Of these, 615 were laboratory-confirmed as DENV-positive based on NS1 antigen detection, anti-DENV IgM detection, and/or RT-PCR. For immunological analyses, 350 samples classified as secondary infections based on an in-house anti-DENV IgG ELISA (IgG titer ≥ 29,000) were included. Among these, 80 samples were successfully serotyped by RT-PCR, comprising 28 DENV-1 and 52 DENV-2 infections, and were included in the serotype-specific immunological analyses. Separately, complete envelope (E) gene sequences were successfully obtained from 32 DENV-1 and 37 DENV-2-positive samples that had been serotyped by RT-PCR and selected from the laboratory-confirmed DENV-positive cases for phylogenetic analysis.

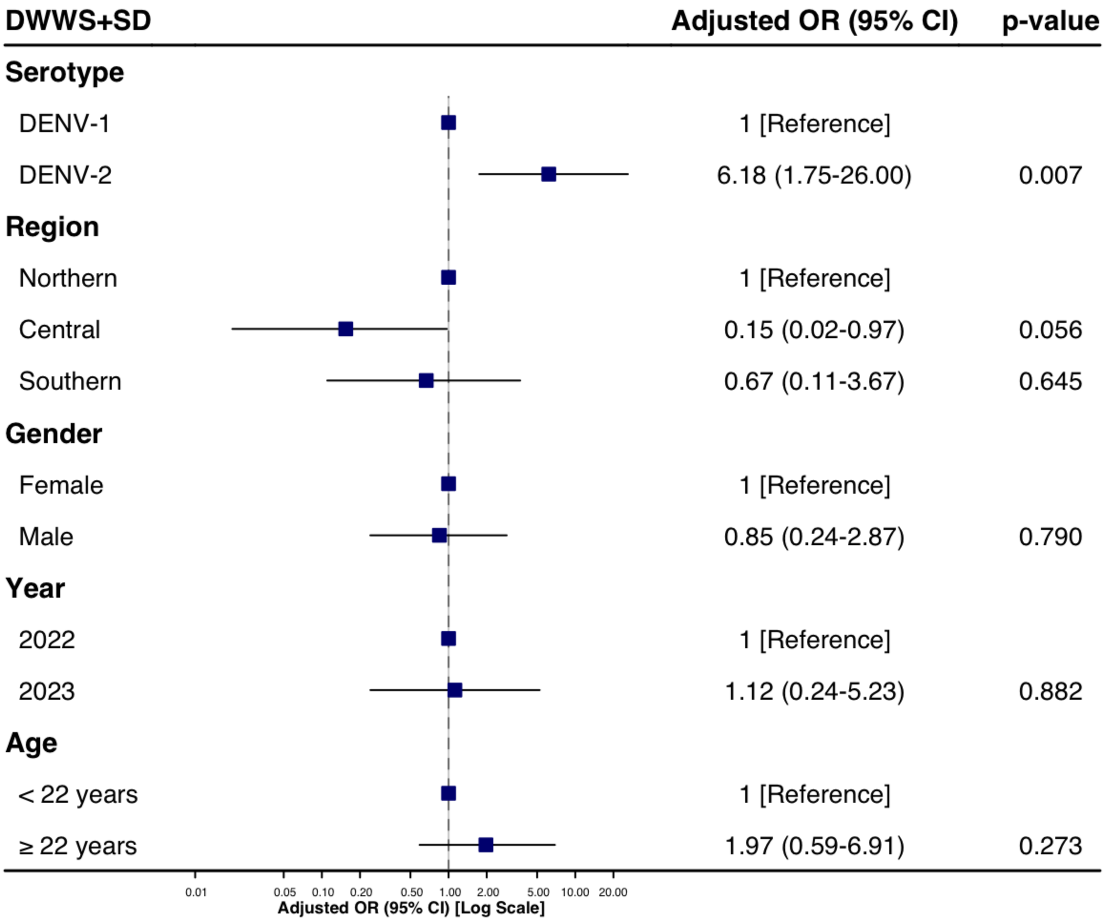

Figure S3. Forest plot of risk factors associated with disease severity. An exploratory multivariable logistic regression analysis was performed to assess the associations between potential risk factors—including DENV serotype, region, sex, year of infection, and age—and disease severity. Dengue without warning signs (DWoWS) was used as the reference category and was compared with dengue with warning signs (DWWS) or severe dengue (SD). The reference groups were DENV-1 (vs. DENV-2), the Northern region (vs. the Central and Southern regions), female sex (vs. male sex), and 2022 (vs. 2023). Age was categorized as binary variable using the median age as the cutoff: < 22 years (vs. ≥ 22 years). Results are presented as adjusted odds ratios (aORs) with 95% confidence intervals (CIs). A two-sided p-value of < 0.05 was considered statistically significant

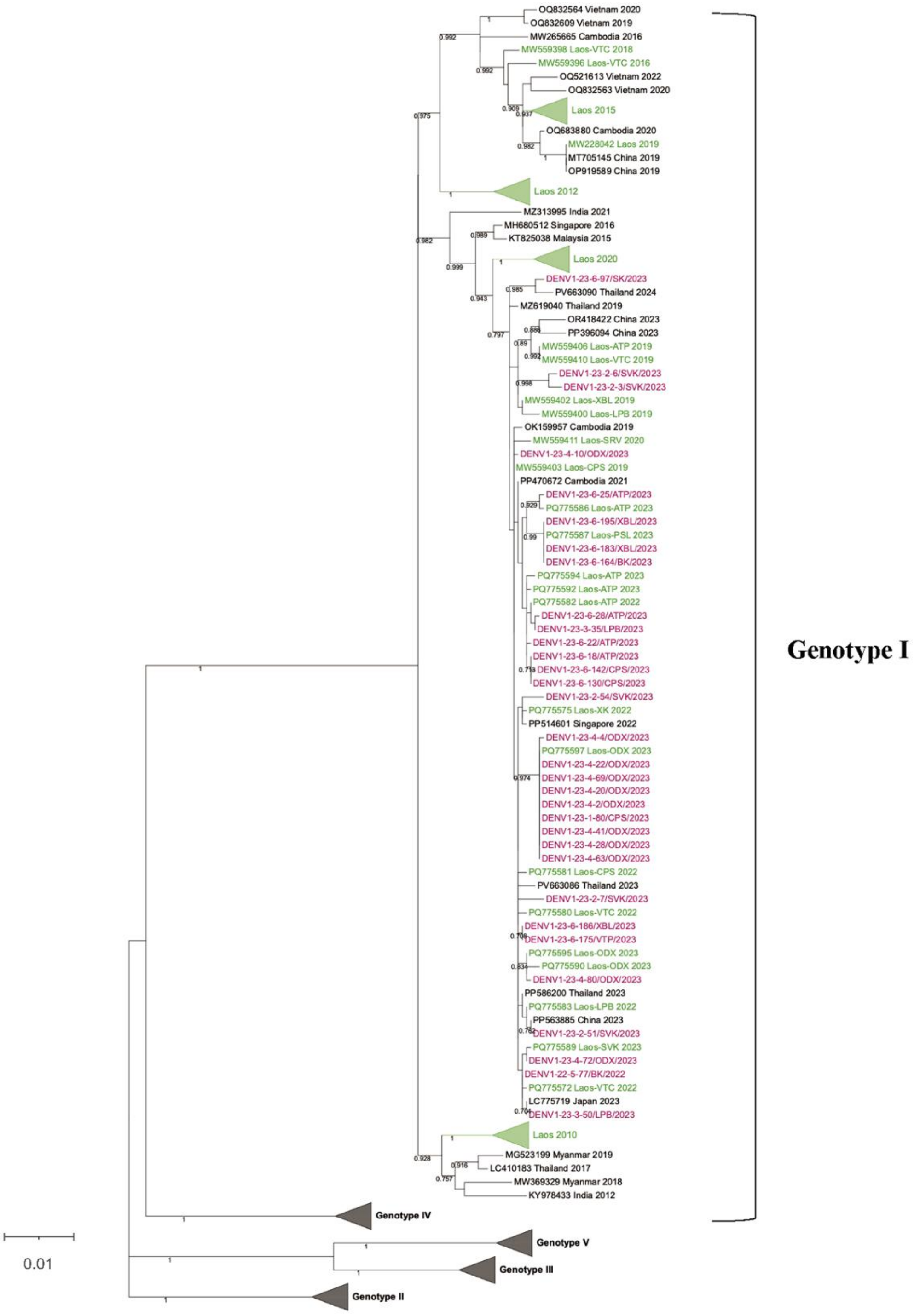

Figure S4. DENV-1 phylogenetic tree and genotypes classification. A maximum-likelihood phylogenetic tree was inferred under the general time reversible model with invariant sites and gamma-distributed rate variation (GTR+I+G) with 1,000 bootstrap replicates. Sequences generated in this study are highlighted in pink, whereas previously published sequences from Lao PDR are shown in green.

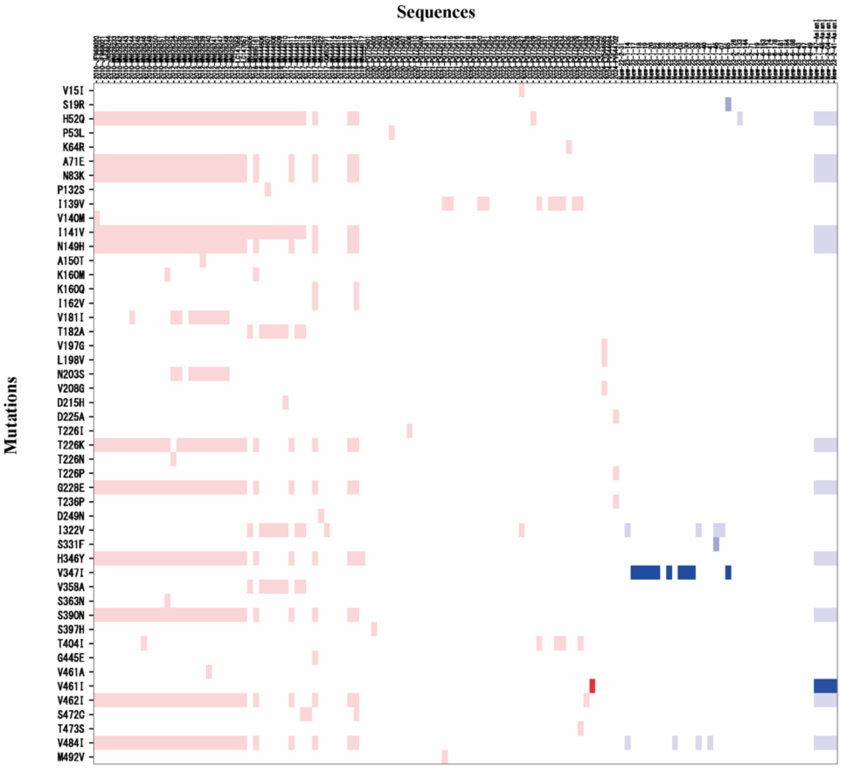

Mutation	New Mut	New No_Mut	Old Mut	Old No_Mut	*p*-value
V15I	1	88	0	37	1.00
S19R	0	89	1	36	0.29
H52Q	40	49	5	32	1.00
P53L	1	88	0	37	1.00
K64R	1	88	0	37	1.00
A71E	31	58	4	33	1.00
N83K	31	58	4	33	1.00
P132S	1	88	0	37	1.00
I139V	10	79	0	37	1.00
V140M	1	88	0	37	1.00
I141V	39	50	4	33	1.00
N149H	31	58	4	33	1.00
A150T	1	88	0	37	1.00
K160M	2	87	0	37	1.00
K160Q	2	87	0	37	1.00
I162V	2	87	0	37	1.00
V181I	10	79	0	37	1.00
T182A	8	81	0	37	1.00
V197G	1	88	0	37	1.00
L198V	1	88	0	37	1.00
N203S	9	80	0	37	1.00
V208G	1	88	0	37	1.00
D215H	1	88	0	37	1.00
D225A	1	88	0	37	1.00
T226I	1	88	0	37	1.00
T226K	30	59	4	33	1.00
T226N	1	88	0	37	1.00
T226P	1	88	0	37	1.00
G228E	31	58	4	33	1.00
T236P	1	88	0	37	1.00
D249N	1	88	0	37	1.00
I322V	10	79	4	33	0.64
S331F	0	89	1	36	0.29
H346Y	32	57	4	33	1.00
V347I	0	89	10	27	1.81e-06
V358A	8	81	0	37	1.00
S363N	1	88	0	37	1.00
S390N	31	58	4	33	1.00
S397H	1	88	0	37	1.00
T404I	5	84	0	37	1.00
G445E	1	88	0	37	1.00
V461A	1	88	0	37	1.00
V461I	1	88	4	33	0.03
V462I	32	57	4	33	1.00
S472C	3	86	0	37	1.00
T473S	1	88	0	37	1.00
V484I	31	58	8	29	0.96
M492V	1	88	0	37	1.00

Figure S5. Comparison of mutations in the DENV-2 E gene between newly sequenced and previously reported Lao-derived strains. Thirty-seven newly sequenced DENV-2 E gene sequences (blue) were compared with 89 previously reported DENV-2 E-gene sequences from Lao PDR (pink). The y-axis indicates the positions of amino acid substitutions, and the x-axis represents individual sequences. Statistical significance was assessed using Fisher's exact test. Mut: mutation; No. Mut: no mutation.

Table S1. List of DENV-1 and DENV-2 envelope (E) gene sequences generated in this study (2022-2023)

Sample ID	Location	Month-year of collection	Serotype	Genotype	Isolation source	GenBank Accession Number
22-5-77	Bokeo, Lao PDR	Oct-2022	DENV-1	I	Serum	PZ177878
23-1-80	Champasack, Lao PDR	Jun-2023	DENV-1	I	Serum	PZ177879
23-2-03	Savannakhet, Lao PDR	Apr-2023	DENV-1	I	Serum	PZ177880
23-2-51	Savannakhet, Lao PDR	Jul-2023	DENV-1	I	Serum	PZ177881
23-2-54	Savannakhet, Lao PDR	Jul-2023	DENV-1	I	Serum	PZ177882
23-2-06	Savannakhet, Lao PDR	Jun-2023	DENV-1	I	Serum	PZ177883
23-2-07	Savannakhet, Lao PDR	Jun-2023	DENV-1	I	Serum	PZ177884
23-3-35	Luangprabang, Lao PDR	Jul-2023	DENV-1	I	Serum	PZ177885
23-3-50	Luangprabang, Lao PDR	Jul-2023	DENV-1	I	Serum	PZ177886
23-4-10	Oudomxay, Lao PDR	Jun-2023	DENV-1	I	Serum	PZ177887
23-4-02	Oudomxay, Lao PDR	Jun-2023	DENV-1	I	Serum	PZ177888
23-4-20	Oudomxay, Lao PDR	Jun-2023	DENV-1	I	Serum	PZ177889
23-4-22	Oudomxay, Lao PDR	Jun-2023	DENV-1	I	Serum	PZ177890
23-4-28	Oudomxay, Lao PDR	Jul-2023	DENV-1	I	Serum	PZ177891
23-4-04	Oudomxay, Lao PDR	Jun-2023	DENV-1	I	Serum	PZ177892
23-4-41	Oudomxay, Lao PDR	Jul-2023	DENV-1	I	Serum	PZ177893
23-4-63	Oudomxay, Lao PDR	Jul-2023	DENV-1	I	Serum	PZ177894
23-4-69	Oudomxay, Lao PDR	Jul-2023	DENV-1	I	Serum	PZ177895
23-4-72	Oudomxay, Lao PDR	Jul-2023	DENV-1	I	Serum	PZ177896
23-4-80	Oudomxay, Lao PDR	Jul-2023	DENV-1	I	Serum	PZ177897
23-6-130	Champasack, Lao PDR	Apr-2023	DENV-1	I	Serum	PZ177898
23-6-142	Champasack, Lao PDR	Jun-2023	DENV-1	I	Serum	PZ177899
23-6-164	Bokeo, Lao PDR	Jul-2023	DENV-1	I	Serum	PZ177900
23-6-175	Vientiane province, Lao PDR	Aug-2023	DENV-1	I	Serum	PZ177901
23-6-18	Attapeu, Lao PDR	Jan-2023	DENV-1	I	Serum	PZ177902
23-6-183	Xayabouri, Lao pDR	Jun-2023	DENV-1	I	Serum	PZ177903

23-6-186	Xayabouri, Lao pDR	Jun-2023	DENV-1	I	Serum	PZ177904
23-6-195	Xayabouri, Lao pDR	Jul-2023	DENV-1	I	Serum	PZ177905
23-6-22	Attapeu, Lao PDR	Jun-2023	DENV-1	I	Serum	PZ177906
23-6-25	Attapeu, Lao PDR	Jun-2023	DENV-1	I	Serum	PZ177907
23-6-28	Attapeu, Lao PDR	Jun-2023	DENV-1	I	Serum	PZ177908
23-6-97	Sekong, Lao PDR	Jul-2023	DENV-1	I	Serum	PZ177909
22-2-31	Savannakhet, Lao PDR	Aug-2022	DENV-2	Cosmopolitan	Serum	PZ178842
23-1-14	Champasack, Lao PDR	May-2023	DENV-2	Cosmopolitan	Serum	PZ178843
23-1-17	Champasack, Lao PDR	May-2023	DENV-2	Cosmopolitan	Serum	PZ178844
23-1-18	Champasack, Lao PDR	May-2023	DENV-2	Cosmopolitan	Serum	PZ178845
23-1-19	Champasack, Lao PDR	May-2023	DENV-2	Cosmopolitan	Serum	PZ178846
23-1-20	Champasack, Lao PDR	May-2023	DENV-2	Cosmopolitan	Serum	PZ178847
23-1-22	Champasack, Lao PDR	Jun-2023	DENV-2	Cosmopolitan	Serum	PZ178848
23-1-23	Champasack, Lao PDR	Jun-2023	DENV-2	Cosmopolitan	Serum	PZ178849
23-1-28	Champasack, Lao PDR	Jun-2023	DENV-2	Cosmopolitan	Serum	PZ178850
23-1-29	Champasack, Lao PDR	Jun-2023	DENV-2	Cosmopolitan	Serum	PZ178851
23-1-03	Champasack, Lao PDR	May-2023	DENV-2	Cosmopolitan	Serum	PZ178852
23-1-30	Champasack, Lao PDR	Jun-2023	DENV-2	Cosmopolitan	Serum	PZ178853
23-1-33	Champasack, Lao PDR	Jun-2023	DENV-2	Cosmopolitan	Serum	PZ178854
23-1-39	Champasack, Lao PDR	Jun-2023	DENV-2	Cosmopolitan	Serum	PZ178855
23-1-40	Champasack, Lao PDR	Jun-2023	DENV-2	Cosmopolitan	Serum	PZ178856
23-1-41	Champasack, Lao PDR	Jun-2023	DENV-2	Cosmopolitan	Serum	PZ178857
23-1-46	Champasack, Lao PDR	Jun-2023	DENV-2	Cosmopolitan	Serum	PZ178858
23-1-47	Champasack, Lao PDR	Jun-2023	DENV-2	Asian I	Serum	PZ178859
23-1-48	Champasack, Lao PDR	Jun-2023	DENV-2	Asian I	Serum	PZ178860
23-1-07	Champasack, Lao PDR	May-2023	DENV-2	Cosmopolitan	Serum	PZ178861
23-1-85	Champasack, Lao PDR	Jun-2023	DENV-2	Cosmopolitan	Serum	PZ178862
23-2-126	Savannakhet, Lao PDR	Oct-2023	DENV-2	Cosmopolitan	Serum	PZ178863
23-2-133	Savannakhet, Lao PDR	Oct-2023	DENV-2	Cosmopolitan	Serum	PZ178864
23-2-144	Savannakhet, Lao PDR	Nov-2023	DENV-2	Cosmopolitan	Serum	PZ178865

23-2-04	Savannakhet, Lao PDR	May-2023	DENV-2	Asian I	Serum	PZ178866
23-2-41	Savannakhet, Lao PDR	Jul-2023	DENV-2	Asian I	Serum	PZ178867
23-2-71	Savannakhet, Lao PDR	Jul-2023	DENV-2	Cosmopolitan	Serum	PZ178868
23-4-19	Oudomxay, Lao PDR	Jun-2023	DENV-2	Cosmopolitan	Serum	PZ178869
23-6-163	Bokeo, Lao PDR	Jul-2023	DENV-2	Cosmopolitan	Serum	PZ178870
23-6-174	Vientiane province, Lao PDR	Aug-2023	DENV-2	Cosmopolitan	Serum	PZ178871
23-6-178	Khammouan, Lao PDR	Jun-2023	DENV-2	Cosmopolitan	Serum	PZ178872
23-6-181	Khammouan, Lao PDR	Jun-2023	DENV-2	Cosmopolitan	Serum	PZ178873
23-6-184	Xayabouri, Lao PDR	Jun-2023	DENV-2	Cosmopolitan	Serum	PZ178874
23-6-188	Xayabouri, Lao PDR	Jun-2023	DENV-2	Cosmopolitan	Serum	PZ178875
23-6-43	Saravane, Lao PDR	Mar-2023	DENV-2	Cosmopolitan	Serum	PZ178876
23-6-47	Saravane, Lao PDR	Mar-2023	DENV-2	Cosmopolitan	Serum	PZ178877
23-6-49	Saravane, Lao PDR	Mar-2023	DENV-2	Cosmopolitan	Serum	PZ178878

S2. Supplementary Methods

S2.1. Statistical Analysis

Out of 350 samples, 55 were excluded because the clinical diagnosis was undefined. DENV serotype data were available for 80 samples; among these 18 lacked clinical severity classification and 1 lacked age data. Consequently, the final analytical subset comprised 61 samples.

An exploratory multivariable logistic regression model was developed to evaluate associations of DENV serotype, region, sex, year of infection, and age with disease severity. The outcome was categorized as dengue without warning signs (DWoWS; reference category) versus dengue with warning signs or severe dengue (DWWS + SD). Because of the small number of cases classified as severe dengue, DWWS and SD were combined into a single category. The reference categories for the categorical covariates were DENV-1 (versus DENV-2), the Northern region (versus the Central and Southern regions), female sex (versus male sex), and 2022 (versus 2023). Age was categorized as a binary variable using the median age as the cutoff: < 22 years versus ≥ 22 years. Sex, age, and year of infection were forced into the model as a priori covariates. DENV serotype and region were then evaluated using forward selection with a predefined inclusion threshold of $p < 0.10$. Candidate variables were retained based on the likelihood ratio test (LRT). All multivariable regression results are presented as adjusted odds ratios (aORs) with 95% confidence intervals (CIs). Statistical significance was defined as two-sided p value < 0.05. Analyses were performed using

R version 4.4.1 (R Foundation for Statistical Computing, Vienna, Austria; https://www.r-project.org).
